# Design and Simulation of an Integrated Wireless Capacitive Sensors Array for Measuring Ventricular Pressure

**DOI:** 10.3390/s18092781

**Published:** 2018-08-24

**Authors:** Natiely Hernández-Sebastián, Daniela Díaz-Alonso, Francisco Javier Renero-Carrillo, Noé Villa-Villaseñor, Wilfrido Calleja-Arriaga

**Affiliations:** 1 CD-MEMS INAOE, Puebla 72840, Mexico; natiely@inaoep.mx (N.H.-S.); paco@inaoep.mx (F.J.R.-C.); 2 MEMS Department, Center for Engineering and Industrial Development, CIDESI, Queretaro 76125, Mexico; daniela.diaz@cidesi.edu.mx; 3 Department of I. T., Electronics and Control, Advanced Technology Center, CIATEQ A.C., San Luis Potosí 78395, Mexico; noe.villa@ciateq.mx

**Keywords:** RF MEMS, pressure sensor, MEMS resonators, implantable BioMEMS, flexible electronics, touch mode capacitive sensor

## Abstract

This paper reports the novel design of a touch mode capacitive pressure sensor (TMCPS) system with a wireless approach for a full-range continuous monitoring of ventricular pressure. The system consists of two modules: an implantable set and an external reading device. The implantable set, restricted to a 2 × 2 cm^2^ area, consists of a TMCPS array connected with a dual-layer coil, for making a reliable resonant circuit for communication with the external device. The capacitive array is modelled considering the small deflection regime for achieving a dynamic and full 5–300 mmHg pressure range. In this design, the two inductive-coupled modules are calculated considering proper electromagnetic alignment, based on two planar coils and considering the following: 13.56 MHz frequency to avoid tissue damage and three types of biological tissue as core (skin, fat and muscle). The system was validated with the Comsol Multiphysics and CoventorWare softwares; showing a 90% power transmission efficiency at a 3.5 cm distance between coils. The implantable module includes aluminum- and polyimide-based devices, which allows ergonomic, robust, reproducible, and technologically feasible integrated sensors. In addition, the module shows a simplified and low cost design approach based on PolyMEMS INAOE^®^ technology, featured by low-temperature processing.

## 1. Introduction

This work addresses a new alternative for measuring blood pressure, using a novel LC sensor arrangement, which can overcome some restrictions that are due to the reduced implantation area available at the left ventricle (LV). Some recent alternatives are still considering the pulmonary artery anatomy dimensions, since it can allow a wider area for the implantation of a more powerful LC radiating inductor [1], however, they are characterized by a limited pressure range [2]. Some biomedical and technology details are described below.

Regarding sensors placed inside the human body for the measurements and wireless transmission of physiological parameters, some cases were proposed since several decades ago. In 1967, Collins [3] developed a passive miniature sensor for the continuous measurement of the intraocular pressure on patients with glaucoma. This device was based on a passive LC resonant circuit, in which the resonant frequency was varied according to the embedded pressure surrounding the device. The electromagnetic coupling of the sensor to an external loop allowed for the wireless transmission, hence determining the resonant frequency of the LC sensor. Then, and considering some suitable calibration, that sensor was able to read the embedding pressure. Starting from that work and with the current advances in microelectronics and microelectromechanical systems (MEMS), several groups began conduct research based on the same principle [4,5,6,7,8,9,10,11,12]. As was evidenced, the available area for the LC array versus power transmission is the main issue to be solved for this kind of implantable sensors, calling for technological improvements in order to meet the implantation requirements.

Blood pressure problems are a kind of disease that chronically damages the blood vessels, organs and tissues of the human body. Public information shows that at least 10% of the world population suffers from these diseases, with the high blood pressure being the main cause of morbidity and mortality in the world [12,13,14,15]. In the heart, the contractions of the ventricular chambers, left and right, provide the force to send the blood to the human limbs, however, sometimes the heart cannot provide enough force to send the blood to the whole body [2]. Thus, it is desired to perform some real time pressure measurements directly inside the chambers of the heart. Accordingly, a successful ventricular pressure monitoring is crucial in medical diagnosis on a series of diseases such as heart failure, aortic aneurysms, strokes, arteriosclerosis and renal failure [16,17].

Currently, there are several blood pressure measurement systems; the most common are the non-invasive devices such as air-filled blood-pressure cuffs linked to a sphygmomanometer and the via auscultator sound method [18]. In addition, non-invasive blood pressure measurements lack of accuracy and stability, since they are indirect measurement techniques [17]. On the other hand, current invasive methods are typically used for percutaneous arterial catheter system, which although are very accurate, they inhibit the free movement of the patient and might be unsafe for long-term use due to complications such as trauma to arterial vessels, infection, hemorrhage and difficulty in obtaining access [18,19,20,21].

The development of polymeric materials has represented one of the most significant tools for the medical area and bioengineering research, since the use of new materials has allowed significant advantages for obtaining implantable devices that can work for a long time, besides they also present additional advantages, such as biocompatibility, low weight, mechanical flexibility and the use of minimally invasive implantation techniques.

In 2006, Fonseca et al. [22] described the first flexible wireless pressure sensor for monitoring abdominal aortic aneurysms. This device was fabricated using a flexible polymer and ceramics which incorporated using lamination techniques, in order to implement a passive resonant circuit. Although this work represents one of the first academic contributions about implanted blood pressure monitors with strong consideration for biocompatibility and minimally invasive functionality, the device precision showed limitations by signal drift and the distance of the electromagnetic transmission.

In 2006 [11], began the development of a new class of implantable devices for the control of aortic aneurysms and heart failure. The system was named CardioMEMS^TM^ [23,24,25,26,27], and consists of an implantable pressure sensor, an external communication module and an intravenous supply system designed to deploy the sensor in the pulmonary artery. The battery-less 3.5 × 30 mm device has a wireless range of about 20 cm. The micromachined device was fabricated utilizing two fused silica wafers, electrodeposited inductors, and fusion bonding. Once implanted, the CardioMEMS^TM^ sensor provided hemodynamic data for systolic pulmonary pressure of 15–35 mmHg, diastolic pressure of 8–20 mmHg and a mean of 10–25 mmHg [26,28]. In 2014 this system was approved by FDA and according to the CHAMPION study, the use of this device in patients with heart failure (HF) has allowed for a reduction of hospitalization events which improved the life quality of the patients [28,29].

**Figure 1:**
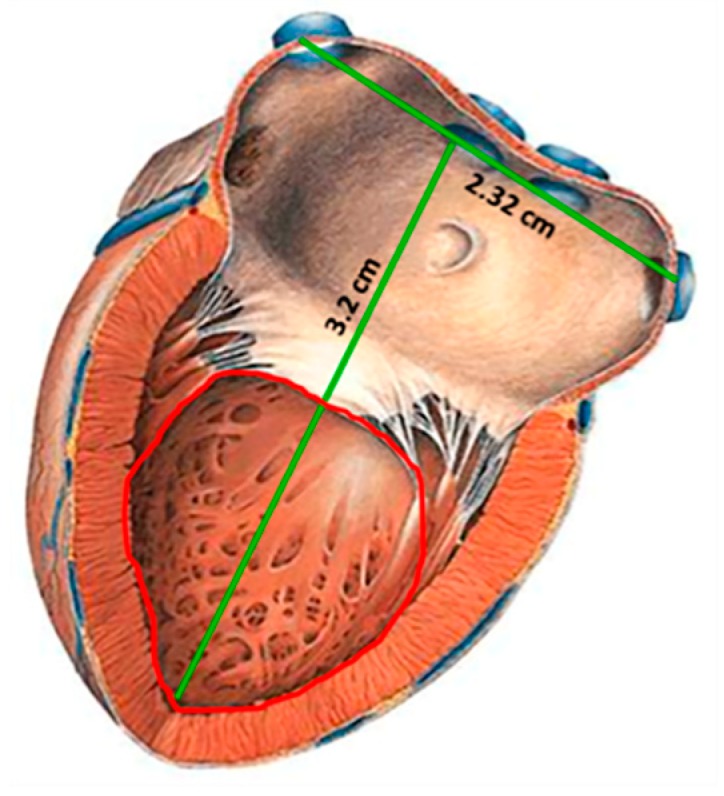
Sketch of the left ventricle [44]; showing the inner section proposed for the sensor implantation.

CardioMEMS^TM^ sensor and most of the pressure sensors designed to be implanted in a place near the heart, are mainly placed in the pulmonary artery (PA), since the pressure in this site can be related to a series of diseases such as heart failure, pulmonary hypertension and aortic aneurysms [19,23,24,25]. In addition, the implantation of pressure sensors in the PA offers a series of design and manufacture advantages such as reduced pressure range (0–80 mm Hg), large devices due to the size of the PA (3 × 3 cm^2^), and the use of techniques for minimally invasive implantation. However, the pressure range measured in PA cannot be related directly to cardiac ventricular contraction and relaxation event. Therefore, if a reliable ventricular pressure sensor can be fabricated and implanted, new diagnostic and therapeutic possibilities could be open, because the LV is the chamber of the heart responsible for pumping the oxygenated blood to the circulatory system [2,29]. As a result, the continuous monitoring of left ventricular pressure, could allow the control of diseases such as: heart failure, hypertrophy in the LV and hypertension; additionally, this will allow the control of secondary diseases such as strokes, renal failure, myocardial infarctions, disease in the coronary artery and aortic aneurysms, placing the sensor permanently in the aneurysmal sac [8,17,19,20,21,30,31,32,33,34,35,36,37,38,39,40,41,42,43]. Figure 1 shows a geometrical approach for the left ventricle, this section is proposed for the sensor implantation, with an inner available area of 2 × 2 cm^2^ [44].

Therefore, an implantable LV pressure device that meets the following considerations is required: wide range of operating pressure (5 to 300 mmHg), small size, appropriated frequency bandwidth, high resolution and precision, biocompatibility and stability in hostile environments. In addition, the sensor design must take into account minimally invasive techniques and anchoring schemes that prevents displacements of the sensor.

**Figure 2:**
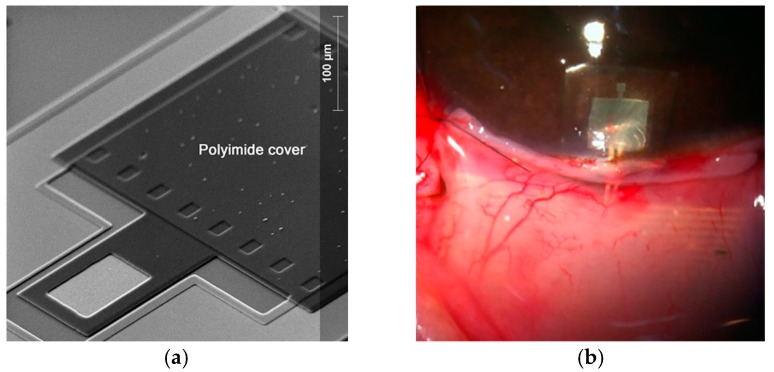
(**a**) Aluminum based capacitive pressure sensor fabricated over a thick polyimide substrate and capped with a thin polyimide film. (**b**) Flexible LC prototype implanted in a rabbit’s eye [45,46,47,48]. Reprinted from Microelectronic Engineering, Vol.159, Félix Gil Carrasco, Daniela Díaz Alonso, Luis Niño-de-Rivera, Biocompatibility and implant of a less invasive intraocular pressure sensor, Pages No. 32-37, Copyright (2016), with permission from Elsevier.

This paper reports the novel design of a two inductive-coupled modules designed for a continuous monitoring of LV pressure. The conception of the implantable capacitive array and the inductive coupling link are designed for accomplish practical, accurate, and real-time wireless pressure sensing. This novel design is supported by our previous work: (a) A magnetically coupled planar coils for wireless power transfer in intraocular pressure measurements [45]; (b) An aluminum based thin film technique for the fabrication of capacitive sensors [46,47], and (c) The implantation of an experimental LC prototype beneath the conjunctiva of a rabbit’s eye using a very simple surgery. The sensors array has the capability to adjust between the conjunctiva and the cornea without an aggressive invasive procedure; the LC array did not suffer rejection; tissue irritation disappears after three weeks; the prototype showed good stability, and the rabbit tolerated this implant during six months before its sacrifice [48]. Figure 2 shows images of this previous work. This implantable sensor is still under fabrication considering a thin-film monolithic approach, defining the capacitive and inductive structures in the same flexible/foldable ergonomic substrate, without the use of hybrid-like connections, combining two manufacturing technologies: surface micromachining and flexible electronics.

The fabrication process was designed according to the PolyMEMS INAOE^®^ technology, which is featured by a low-temperature processing, considering materials for assuring biocompatibility. Finally, this sensor can also be adapted for monitoring the pressure in different organs, such as the aorta, pulmonary artery and even the urinary bladder.

## 2. Integrated Wireless System Description

**Figure 3:**
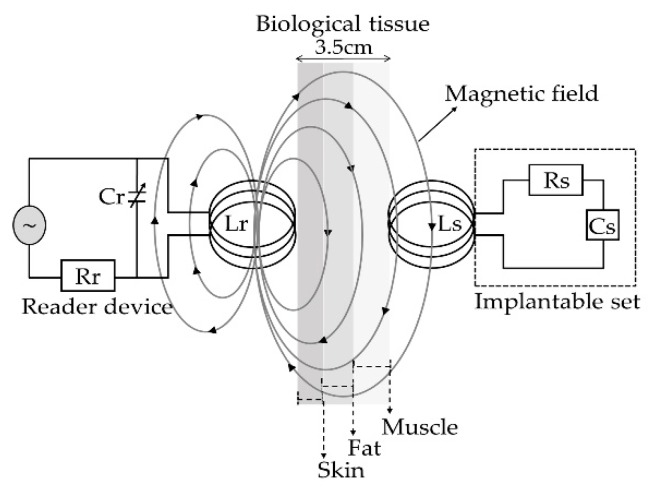
Electromagnetic scheme for the wireless ventricular pressure sensor.

The concept supporting the wireless ventricular pressure sensor, in a passive electrical sensing scheme, is shown in Figure 3. The complete monitoring system consists of two modules: an implantable sensor set and an external reader device. In this design, the two inductive-coupled modules are calculated considering a proper electromagnetic alignment, based on two circular planar coils with the proper resonant frequency, calculated as [49,50,51]:

(1) $$\;f=\frac{1}{2\pi \sqrt{\mathrm{LC}\;}}{\;\mathrm{when}\;R}^{2}\gg \frac{L}{C}$$

where L, C and R denote the magnitudes for inductance, capacitance and resistance, respectively. In addition, for this design both coupled modules are modelled as a multicore transformer for transmission/reception power. That is, when a time varying current circulates though the coil (L_r_) from the reader device, an electromagnetic field is radiated around it. If the coil (L_s_) from the implantable set is inside the radiation zone, some electromagnetic field lines cross the L_s_ area, generating a time varying current on the implantable set and operates according to the C_s_ magnitude. C_s_ will vary following the ventricular pressure, and the proper electromagnetic coupling is the main subject for this work.

The resonant frequency of the implanted sensor set and the signal coupling towards the external coil can be modeled as a two-port network. Under this premise, the input impedance of the reader coil is expressed taking electrical parameters from the implantable device [49,50,52], as follows:

(2) $$\;Z_{\mathrm{eq}\;}=\frac{V_{r}}{I_{r}}\;=\;j2\pi fL_{r}\left[{1\;+\;k}^{2}\frac{{{(f/f}_{s})}^{2}}{{1-\;(f/f}_{s}{)}^{2}{\;+\;(1/Q}_{s}{)j(f/f}_{s})}\right]$$

where V and I are the exciting voltage and current across the reader coil, f is the excitation frequency, k is the coupling factor (totally dependent on physical dimensions), f_s_ is the resonating frequency of the implanted sensor set and Q = (2πf_s_L_s_)/R_s_ is the quality factor of the sensor under resonance.

It can be seen from Equation (2) that in order to change the impedance Z_eq_ from the reader coil, one must change either the k or f_s_ of the implanted set. For ventricular pressure applications, the distance between both coils will remain constant so the k will not change. Therefore, f_s_ is the only parameter capable of changing the equivalent impedance. According to Equation (1), the overall variation of the capacitance into the implanted sensor array is caused by a local change of the pressure, which accordingly changes the resonant frequency. Such changes are detected in the reading coil as variations in the equivalent impedance, and hence related to the ventricular pressure.

### 2.1. Implantable LC Sensor Set

**Figure 4:**
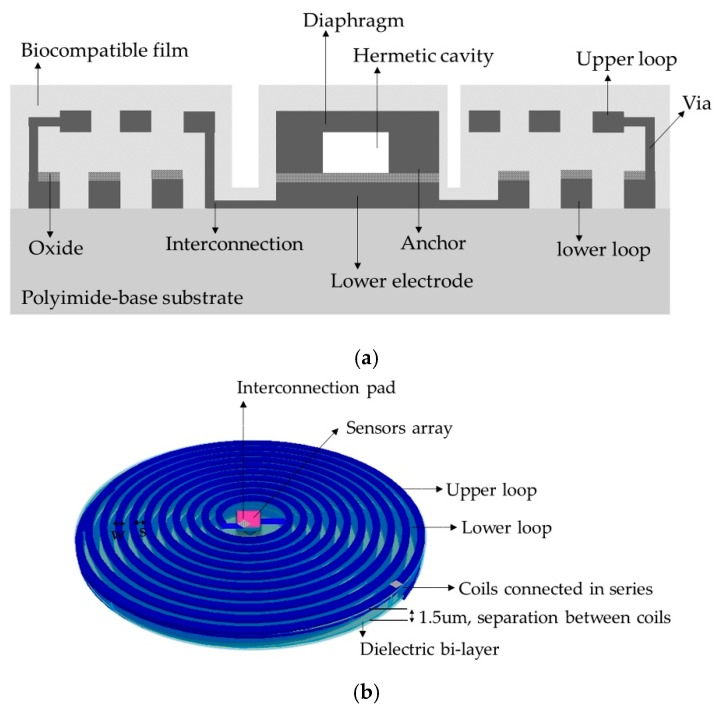
(**a**) Cross section cut of the implantable sensor set, and (**b**) A 3D view.

The implantable sensor set, restricted to an area of 2 × 2 cm^2^ (according to the LV internal dimensions), was defined over a 20 µm-thick polyimide film and interconnected according to Figure 1. It consists of two touch-mode capacitive pressure sensors (TMCPS) arranged in parallel and connected to a dual-layer planar coil; thus, a reliable resonant circuit for communication with the external device is attained. The implantable set was designed considering a thin-film monolithic approach, highlighting the fabrication of both, the capacitive and inductive structures, on the same flexible ergonomic substrate without the use of hybrid-like connections. This was achieved by using the combination of two manufacturing technologies, the surface micromachining and flexible electronics Figure 4 shows a 3D view of the double-layer coil and a cross-section view of the implantable sensor set.

**Figure 5:**
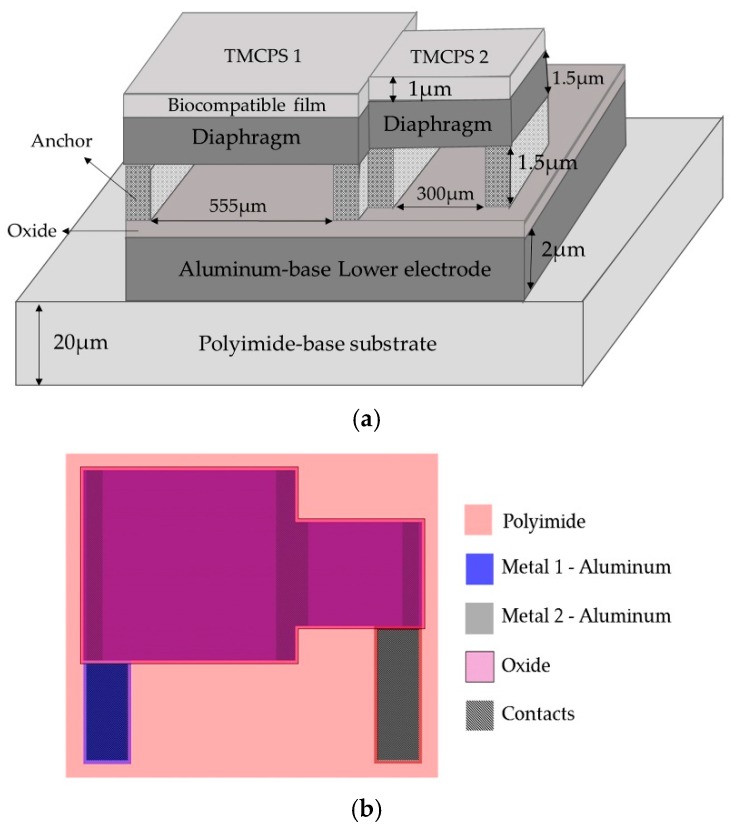
(**a**) Double diaphragm capacitive array, the sketch shows the structural materials and dimensions. (**b**) Layout.

The proposed novel capacitive array is shown in Figure 5a, it consists of a sectioned hermetic chamber with two parallel capacitors array. In this approach, both plates are isolated by a double insulator, air/silicon oxide, allowing a dynamic variable capacitive sensor [47,53,54,55], as can be seen in the layout of Figure 5b. The 555 µm-side capacitor was mechanically designed in order to respond under the lower LV pressure regime, while the 300 μm-side capacitor was designed to get a response under the highest LV pressure regime. This capacitive array was fully designed considering a thin polyimide film added as a biocompatible capping film, which at the same time is part of the diaphragm of the capacitors. Finally, the capacitors were analyzed as follows: (a) the top diaphragm is calculated to provide a direct contact with the physiological environment, thus, the structures were properly covered with a biocompatible film; (b) the double-film squared diaphragm (polyimide over aluminum) was structurally modeled considering the small deflection regime [54,55]. This analytical work concluded with two precise mechanical complementary capacitors, capable of achieving a dynamic 5–300 mmHg pressure range to cover the full diastolic-systolic pressure range developed across the LV [2,19].

The detection principle of the capacitive array is based on the relationship between the changes in capacitances for a given applied pressure [8,47,49,54]. In this case the total capacitance, at any time, is the sum of the individual capacitances associated at a given pressure, as follows:

(3) $$\;C_{s}\;=\;\frac{\varepsilon_{0}\varepsilon_{\mathrm{aire}\;}\varepsilon_{d1}A_{\mathrm{touch}1}}{W_{\max1}{\;+\;\varepsilon }_{d1}W_{\max1}}+\frac{\varepsilon_{0}\varepsilon_{\mathrm{aire}}\varepsilon_{d2}A_{\mathrm{touch}2}}{W_{\max2}{\;+\;\varepsilon }_{d2}W_{\max2}}$$

where ε_d_ is the dielectric constant of the insulating material, A_Touch_ is the contact area of the diaphragm, W_max_ is the separation distance between the parallel metal plates and the subscripts 1 and 2 stand for the first and second capacitive structure, respectively. Table 1 shows the main parametric design and the analytical results for the capacitive array. Both diaphragms were calculated to operate simultaneously based on the minimum (5 mmHg) and the maximum (300 mmHg) operating pressures of the LV, thereby ensuring that the capacitive assembly covers the full range of the ventricular pressure.

**Figure 6:**
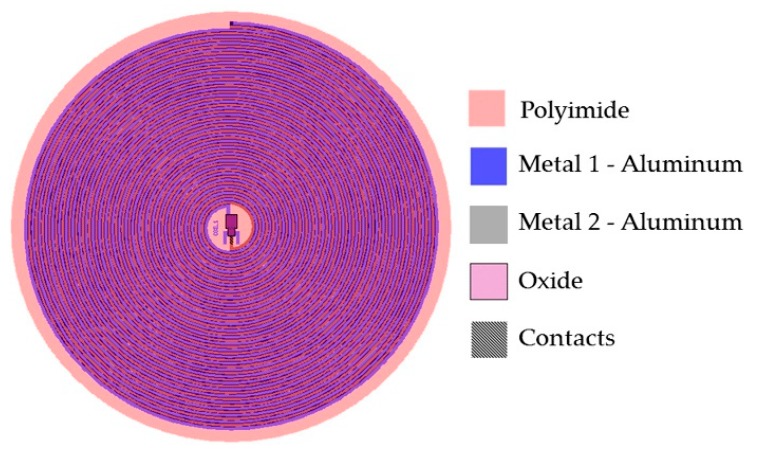
Double layer internal coil layout showing the 5-level design materials.

Due to the restricted area existing inside the LV, the design of the internal coil consisted of a dual-layer planar inductor in order to increase both, the total value of the inductance and its quality factor. The two superposed aluminum loops, isolated by a bi-layer dielectric (oxide and polyimide), were connected in series and each one was composed by 28 turns, presenting an external diameter of 2 cm. Regarding the metal and coil thicknesses, they were chosen taking into account that the full implantable set must facilitate the thin-film monolithic approach. As shown in the layout of Figure 6, the full array is covered by a thin polyimide film.

The electric characteristics of the dual-layer planar coil can be determined by using well known models [56,57,58], where the electrical inductance for a circular multi-layer coil can be calculated as:

(4) $$\;L≅L_{1}{\;+\;L\;}_{2}\;\pm \;2M$$

where M = k(L_1_∙L_2_)^1/2^ is the mutual inductance between the two levels of the planar coil [28], k = (R^2^_out.T_∙R^2^_out.R_)/(R^2^_out.T_∙R^2^_out.R_)^1/2^(R^2^_out.T_ + X^2^)^3/2^ is the coupling factor between the two coils, whereas L_1_ and L_2_ are the self-inductances for the lower and upper loops, which are determined from the following Equation [49,50,57,59]:

(5) $$\;L_{1}{\;=\;L\;}_{2}≅\frac{\mu_{0}n^{2}d_{\mathrm{avg}}C_{1}}{2}\left[\ln\left(\frac{C_{2}}{F}\right){\;+\;C}_{3}{F\;+\;C}_{4}F^{2}\right]$$

where *n* = (Rout − Rin)(w + s) is the number of turns of the inductor, davg = (Dout + Din)/2 is the averaged diameter of the windings, F = (Dout − Din)/Dout + Din) is the fill factor of the windings and C1–C4 are constant coefficients determined by the winding geometry [57].

From Equation (4) we can observe that for a multi-layer system, the final inductance increases according to a positive effective mutual coupling. Figure 6 shows the layout of the sensor set. This design has several advantages that include small size, stability, ergonomic and mechanical flexibility. Additionally, the distribution of the windings is not superposed, in this way there is no contribution to parasitic capacitances, and then the mutual coupling results positive.

### 2.2. External Coil

The external coil was calculated under flexible conditions taking into account the physical dimensions and materials for its manufacturing. It was fabricated on a 4-layer PCB FR-4 as the substrate material, composed by a 27-turns copper coil and 8-cm outside diameter, and designed according to the Finkenzeller condition [52,59]:

(6) $$\;D_{\mathrm{out}.T\;}\;\leq \;D2\sqrt{2}$$

(7) $$\;R_{\mathrm{out}.T\;}\;\geq \;\sqrt{X^{2}{\;+\;R}_{\mathrm{out}.R}^{2}}$$

where D_out.T_ = 2R_out.T_ is the outer diameter of the outer coil, D is the radiation distance and X is the separation between the inner and outer coils.

**Figure 7:**
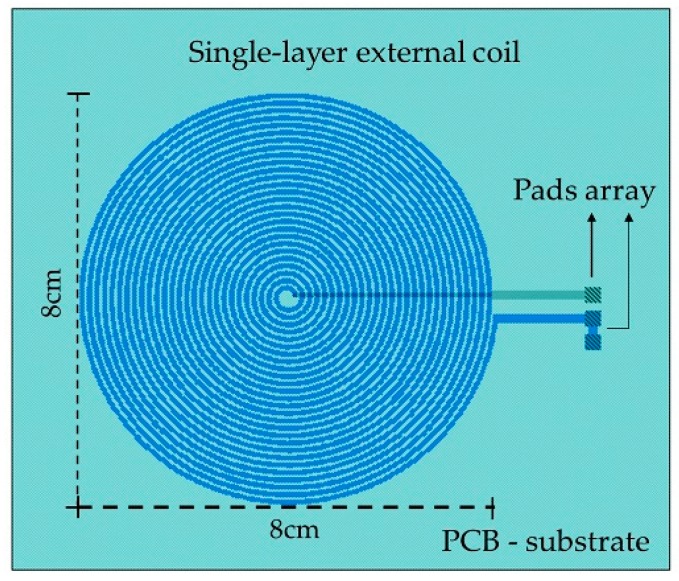
Layout generated for the external coil.

The electromagnetic coupling was calculated considering: (a) 13.56 MHz frequency to avoid tissue damage by radiation and heating (according to ISO 14117 for implantable devices), and (b) in order to simulate a more realistic environment, we considered a core composed by three layers to simulate the biological tissue: skin with a 0.5 cm thickness, fat with a 1 cm thickness, and muscle with a 2 cm thickness. Design parameters such as the number of turns, width, thickness and the value for the inductive element were determined based on the self-inductance value of the implantable sensor set, therefore the two RCL circuits resonate at the same frequency. Figure 7 shows the lay out of the external coil.

The self-inductance of the external and internal coils was calculated based on the number of turns taken from Equation (5). For the inductive coupling link, the electrical parameters were calculated using well known methods [51,59,60], and the power transmission efficiency for the inductive link is given by:

(8) $$\;ƞ\;=\frac{k^{2}Q_{1}Q_{2}^{3}R_{2}R_{\mathrm{load}\;}}{\left(k^{2}Q_{1}Q_{2}^{3}R_{2}R_{\mathrm{load}}{\;+\;k}^{2}Q_{1}Q_{2}R_{\mathrm{load}}^{2}{\;+\;Q}_{2}^{4}R_{2}^{2}{\;+\;2Q}_{2}^{2}R_{2}R_{\mathrm{load}}{\;+\;R}_{\mathrm{load}}^{2}\right)}$$

where Q = (1/R)(L/C)^1/2^ represents the quality factor for the external and internal coils, R_2_ is the equivalent resistance of the internal coil, R_load_ ≥ 2ωL_2_ is the load resistance [59], and for this case R_load_ = 3 kΩ. Table 2 lists the main deign parameters and the analytical results for the internal and external coils.

## 3. Results and Discussion

### 3.1. Capacitive Array

**Figure 8:**
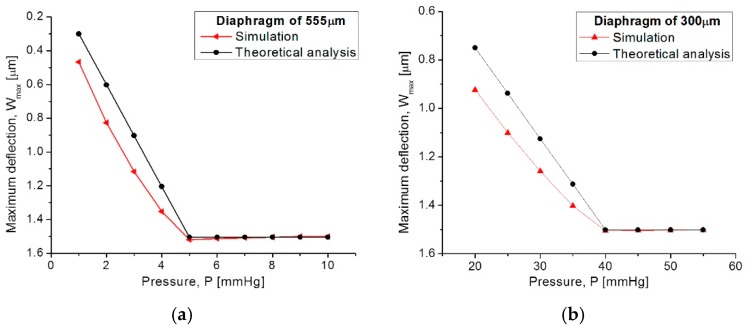
Comparison of analytical and simulated W_max_-P curves obtained from (**a**) the 555 µm-side diaphragm and (**b**) the 300 µm-side diaphragm.

The capacitive array was analyzed using the CoventorWare^®^ software based on the finite element method to evaluate the mechanical deformation of both diaphragms. In addition, the obtained parameters (strain, stress, electrical resistance, and finally the C-P characteristics) and the resultant quantitative curves were used as a design tool to achieve a desired electromechanical performance. Figure 8a compares both, the analytical and simulated diaphragm maximum deflection versus the applied pressure (W_max_-P curves), obtained for the two designed squared capacitive structures: 555 µm- and 300 µm-side. Figure 8b illustrates the initial touching operation pressure (P_Touch_) for each diaphragm.

**Figure 9:**
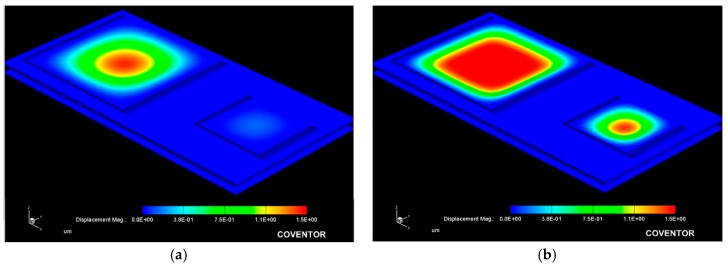
Simulated mechanical response obtained from the capacitive array at a (**a**) 10 mmHg and (**b**) 100 mmHg applied pressure.

Figure 9 allows for a 3D qualitative visualization of the mechanical response under an applied pressure. It can be observed that the large structure operates at the low pressure regime while the small one operates at the high pressure regime.

From Figure 8, the analytical model agrees well the calculated mechanical response, where the maximum deflection (touching pressure) occurs at 5 mmHg and 40 mmHg, according to the size of each squared diaphragm. The slight variation observed at the beginning of the W_max_-P curves are due to the fact that the analytical calculations neglect some deformations at the middle plane of the composed diaphragm, whereas the simulation software recreates the complete trajectory of the diaphragm, considering key structural parameters and a more complex analysis.

**Figure 10:**
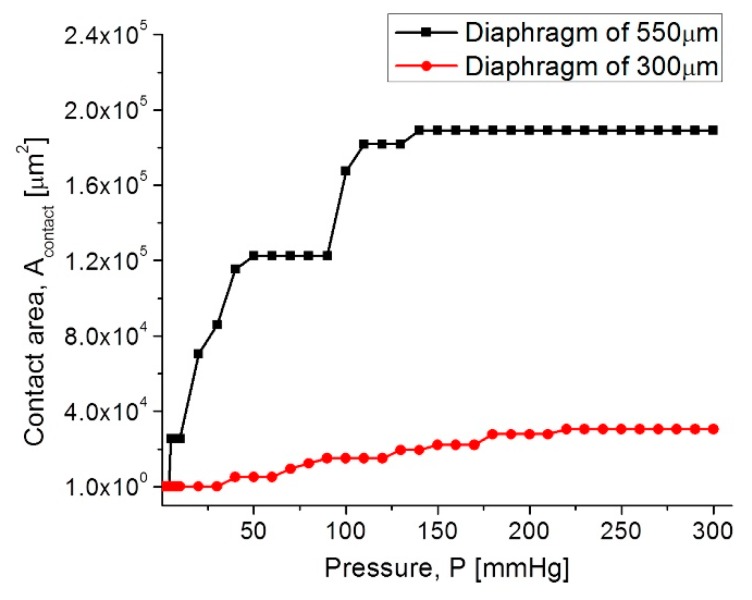
A_contact_-P curves for the capacitive (TMCPS) sensor array.

Once the capacitors (TCMPS) parameters have been determined for achieving an optimum performance, the maximum operating pressure was evaluated for an increasing pressure, which was used to simulate the touching contact area (A_contact_) over the isolated lower plate. Therefore, once the contact area stops increasing, the applied pressure at this point is the maximum operating pressure. Figure 10 shows the A_contact_-P curves, where the maximum operating pressure for each capacitive structure are plotted. The lower pressure regime corresponds to the 550 µm diaphragm, and the higher pressure regime is for the 300 µm diaphragm.

In both diaphragms under an increasing pressure regime, the variations of the touching contact area seem slight, however these variations are high enough to produce significant changes in the total capacitance, and consequently, they produce changes in the resonant frequency of the RCL circuit (see Equation (1)). The touch contact area can be expressed as A_Touch_ = K_1_P − K_2_P^2^, where K_1_ y K_2_ are the linear and saturation constants, respectively, and K_1_ >> K_2_ [53]. Therefore, under a determined pressure, the contact area is proportional to the pressure, and the corresponding capacitance can be determined directly. This is because the overall response is a linear C-P relationship, typical of a touch mode capacitive pressure sensor, which is very suitable for the conditioning circuitry.

**Figure 11:**
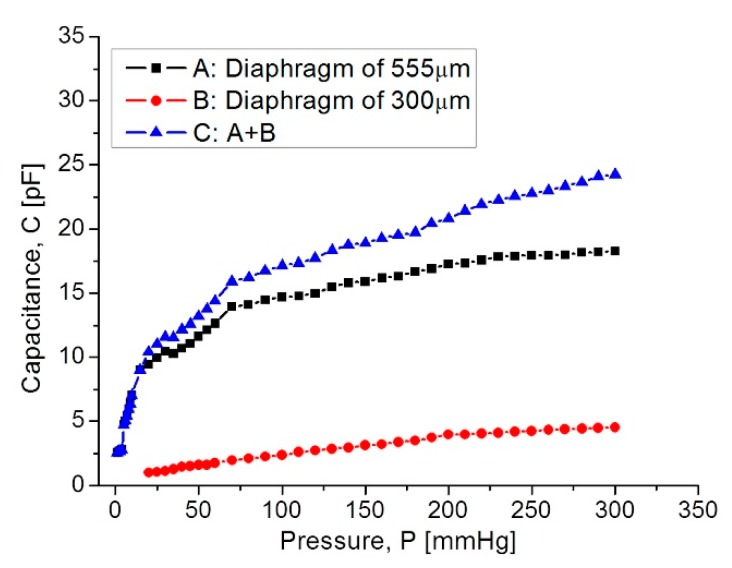
Capacitance response versus applied pressure, covering the full 5–300 mmHg range.

Figure 11 shows the C–P characteristic curves obtained for each as well for the full set of capacitive sensors.

**Figure 12:**
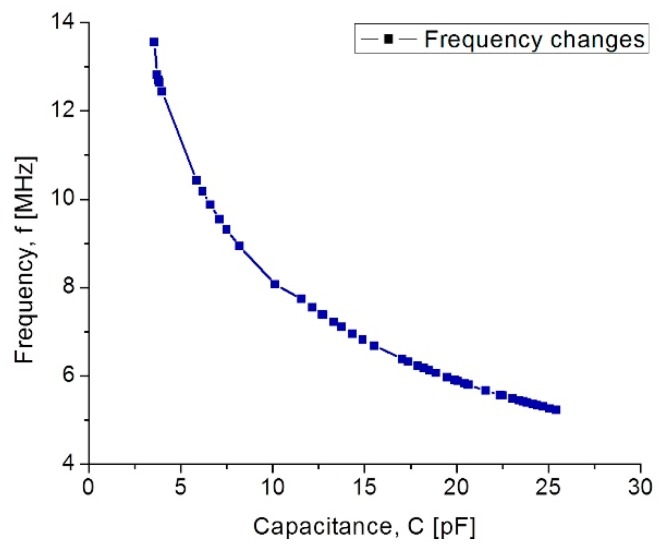
Simulated operating frequency as a function of capacitance for the implantable set.

In Figure 11, curve C shows that at the beginning the capacitance increases suddenly because the 555 µm diaphragm quickly makes contact over the lower electrode. In the 15–75 mmHg range, the capacitance increases with a linear rate typical for the 555 µm diaphragm. Around 80–300 mmHg, the capacitance increases linearly and steadily, influenced by the 300 µm diaphragm according to the simulation routines. This electromechanical response of the capacitive sensors set was analyzed once it was interconnected with the planar coil. As expected, the capacitance variations led to changes on the resonance frequency. Figure 12 shows the simulated operating frequency versus the capacitance variations, according to the circuit shown in Figure 3.

According to simulations, the operating frequency for the implantable sensor set presents a variation from 13.56 MHz to 5.2 MHz; this frequency range which is included within the industrial, medical and scientific band (ISM), and completely ensures the safety because no tissue damage by radiation can occur. Another key parameter for analyzing the capacitive pressure sensor is the sensitivity ΔC/ΔP as a function of frequency.

**Figure 13:**
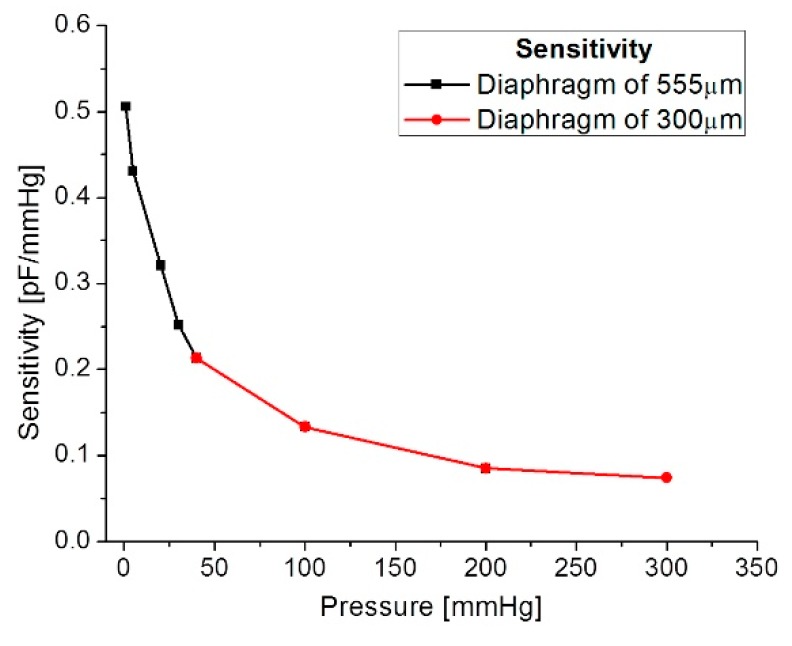
Sensitivity versus applied pressure.

Our mathematical analysis and simulations make evident some changes in the sensor sensitivity, considering the operating frequency range. Figure 13 shows the relationship between the sensitivity and the applied pressure, considering each capacitive structure. As observed, the sensitivity decreases when the applied pressure increases. This sensitivity is influencing the final power transmission efficiency to be discussed later.

### 3.2. Inductive Coupling Link

The overall inductive coupling link was modeled considering three main factors (internal dual-layer coil, external coil and protocol coupling link), using the Comsol Multiphysics^®^ software considering the physical interfaces. Each factor was modeled explicitly and with a homogenized approach for obtaining several parameters, such as the self-inductance (L), mutual inductance (M), electric resistance (R), magnetic flux density (Φ), and the induced current (i).

#### 3.2.1. Internal Dual-Layer Coil Model

**Figure 14:**
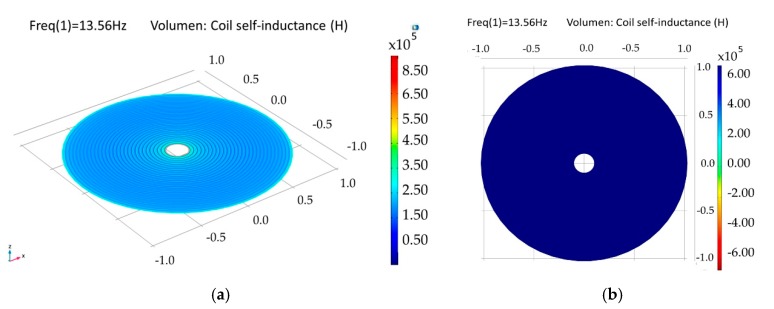
Simulation results for the internal double-level coil. (**a**) Self-inductance for the composed double coil and (**b**) self-inductance for a single-element coil.

The internal dual-layer coil was simulated by parts and as a single-element, because a multilevel coil involves more coupling factors than those needed for a single-layer coil (see Equation (4)). As a composed-element, a self-inductance of 6.68 µH was obtained for each loop, and an electric resistance of 77 Ω and 158 Ω were obtained for the lower and upper loop, respectively. For the single-element coil, separated 1.5 µm by a dielectric material (polyimide) and planarly oriented, a coupling factor of 0.99, a self-inductance of 27.1 µH and an electric resistance of 259 Ω were obtained; this is shown in Figure 14.

The model for a dual-layer planar coil implemented in the implantable set, allowed for obtaining higher values of inductance in a small area (restricted by the anatomical dimensions of the LV, which results in better characteristics in terms of the internal coupling factor and power transmission efficiency. Table 3 shows the simulation results for the internal coil.

#### 3.3.2. External Coil

The external coil was simulated in a similar way to the internal coil, however, a less complex system was considered, since the coil is formed by a single loop, thus only the explicit simulation model was used. A self-inductance of 19.7 µH, a quality factor of 512 and an electric resistance of 5.6 Ω were obtained.

#### 3.3.3. Magnetic Coupling Link Model

**Figure 15:**
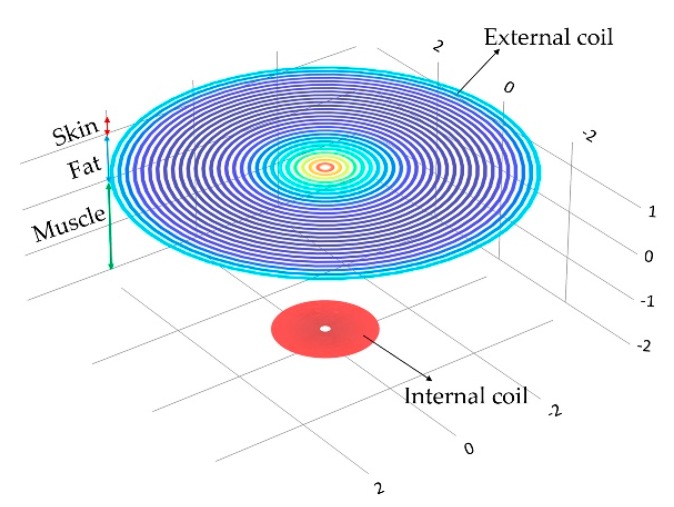
Inductive coupling link across biological tissue.

The mathematical analysis of the inductive coupling link was validated with the Comsol Multiphysics software based on the near field approximation. The simulation model considers that the external coil is located outside the human body but establishes communication across the surface skin, where the internal coil is mounted within the left ventricle at a depth of 3.5 cm. In addition, we considered a 13.56 MHz resonance frequency to avoid tissue damage by radiation and heating, and in order to simulate a more realistic coupling environment, the core considered three types of biological tissue: skin, fat and muscle, as shown in Figure 15. Table 4 shows the parameters used for the composed biological tissue [61,62].

**Figure 16:**
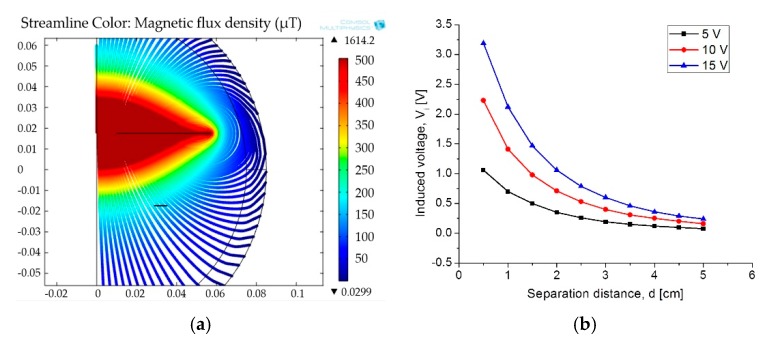
(**a**) Magnetic flux density and (**b**) induced voltage changes as a function of the separation distance.

For medical applications, a key factor is the inductive coupling link because part of the field dissipates in the tissue leading to some power dissipation. It is clear that as the distance between the coils decreases, the electromagnetic field density increases, as well as other parameters such as the mutual inductance, the induced current/voltage, and the power transmission efficiency. Figure 16 shows the magnetic field density of the inductive coupling link and the relationship between the distance separating the coils and the induced voltage for an input voltage of 5 V, 10 V and 15 V.

**Figure 17:**
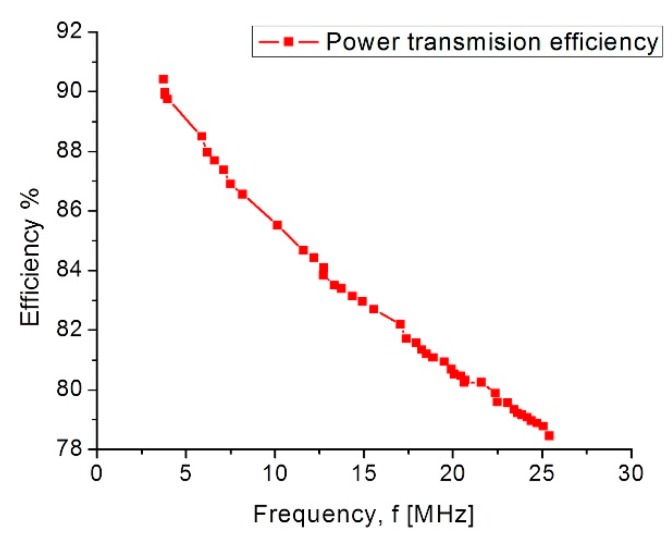
Power transmission efficiency versus frequency.

Finally, the simulation results for the coupling across. The biological tissue delivered the following results: first, it showed a 90% power transmission efficiency under the lower pressure range; second, under the higher pressure range the efficiency decreased to 78%. This controlled coupling attenuation comes from the smooth capacitance transition on the TMCPS array; Figure 17 shows this relationship.

Table 5 list the simulation parameters obtained for the inductive coupling link, which are in good agreement with the theoretical analysis.

## 4. Conclusions

We report the design of a new sensor scheme capable of measuring ventricular blood pressure, which will allow for the continuous monitoring of some diseases such as heart failure, aortic aneurysms and hypertension. The novel implantable sensor design, composed by capacitive and inductive structures, was arranged on the same flexible substrate, avoiding hybrid-like connections, and combining both manufacturing technologies, surface micromachining and flexible electronics. The capacitive sensors array was designed using a composed aluminum/polyimide diaphragm, where the structure and its parallel interconnection are arranged in order to cover the wide LV pressure range, which is a key contribution of this work. According to the anatomy of the LV, an internal dual-layer coil was implemented in order to increase the L and Q parameters in a reduced physical area. The model for a dual-layer planar coil allows for obtaining better characteristics in terms of the internal coupling factor and the power transmission efficiency. The simulation set delivered the following figures of merit: a full dynamic 5–300 mmHg pressure range, and an operating frequency range of 5.2–13.56 MHz. This proposal complies with the full diastolic-systolic pressure range developed across the LV, which follows the ISO 14117 standard for implantable devices and the industrial, medical and scientific band (ISM). Currently the fully integrated process fabrication is under progress.

The electromagnetic coupling across the biological tissue was validated with the Comsol Multiphysics software: first, it showed a 90% power transmission efficiency, for a 3.5 cm separation between coils, under the lower pressure range; second, under the higher pressure range the efficiency decreases to 78%. Concerning the module fabrication, and according to our previous experimental work, we modified the PolyMEMS INAOE^®^ technology, for an aluminum-based technique, which allows for obtaining ergonomic, robust, reproducible, low-cost, and technologically feasible inductive and capacitive structures. The polyimide substrate and coating contribute for reducing the tissue damage and also offers a minimally invasive implantation procedure. This dynamic array is designed considering a biocompatible dual-layer diaphragm and a low temperature fabrication approach, such as the PolyMEMS INAOE^®^ technology, but these novel interconnected microstructures could be fabricated using some other technology if the microelectromechanical parameters are fulfilled. Finally, this sensor can also be adapted for monitoring the pressure in different organs such as the aorta, pulmonary artery and the urinary bladder.

## Figures and Tables

**Table 1:** Design parameters and analytical results for the capacitive array.

Parameters	Symbol	Structure 1	Structure 2
Contact pressure	P_Tocuh_	5 mmHg	40 mmHg
Maximum operating pressure	P_Max_	200 mmHg	300 mmHg
Lateral length of the diaphragm	a	555 µm	300 µm
Thickness of the lower electrode	t_Elow_	2 µm	2 µm
Thickness of the top electrode	t_Etop_	1 µm	1 µm
Thickness of the biocompatible film	t_Bio_	1.5 µm	1.5 um
Air gap	W_max_	1.5 µm	1.5 µm
Oxide thickness	t_oxi_	0.2 µm	0.2 µm
Sensor capacitance at zero pressure	C_p0_	1.9 pF	0.51 pF

**Table 2:** Analytical results and design parameters for the internal and external coils.

Quantity	Symbol	Internal Coil	External Coil
Internal diameter	Din	2 mm	2 mm
External diameter	Dout	2 cm	8 cm
Width of the metal lines	w	160 µm	700 µm
Space between turns	s	160 µm	700 µm
Thickness of the metal lines	h	2 µm y 1 µm	35 µm
Number of turns	N	28 each loop	27
Length	l	1.14 m	1.7 m
Operating frequency	fs	13.56 MHz	
Self-Inductance	L	20.05 µH	21.29 µH
Electric resistance	R	309 Ω	4 Ω
Quality factor	Q	8	591
Load resistance	Rload	3 kΩ	---
Radiation distance	X	3.5 cm	
Coupling coefficient	k	0.054	
Mutual inductance	M	2.5 µH	
Power transmission efficiency	η	90%	

**Table 3:** Simulation results for the internal dual coil.

Parameters	Lower Loop	Upper Loop	Binding
Electrical resistance	77 Ω	158 Ω	259 Ω
Self-inductance	6.68 µH	6.68 µH	21.12 µH
Quality Factor	17	8.3	9.1
Internal coupling factor ^1^	0.99		
Internal mutual inductance ^1^	6.67 µH		

^1^ Parameters between the lower and upper loops.

**Table 4:** Constitutive parameters of human biological tissue at a frequency of 13.53 MHz.

Model	Thickness (cm)	Conductivity (Sm^−1^)	Relative Permittivity	Wavelength (m)
Dry skin	0.5	0.23802	285.25	2.26
Wet skin		0.38421	177.13	2.87
Fat	1	0.030354	11.827	11.11
Muscle	2	0.62818	138.44	3.24

**Table 5:** Simulation parameters for the inductive coupling link.

Parameters	Symbol	Value
Resonance frequency	$f_{s}$	13.56 MHz
Mutual inductance	M	3.38 µH
Magnetic flux density	∅	150 µT
Coupling efficiency	k	0.054
Radiation distance	X	3.5 cm
Power transmission efficiency	ɳ	90.7%
